# Early serum screening for hepatocellular-carcinoma in patients with hepatitis

**DOI:** 10.1186/1471-2334-14-S7-P28

**Published:** 2014-10-15

**Authors:** Alina Cristina Neguț, Anca Streinu-Cercel, Oana Săndulescu, Mădălina Carap, Mihaela Adriana Toderici, Adrian Streinu-Cercel

**Affiliations:** 1 Carol Davila University of Medicine and Pharmacy, Bucharest, Romania; 2 National Institute for Infectious Diseases "Prof. Dr. Matei Balş", Bucharest, Romania; 3 Lotus-MED Medical Center, Bucharest, Romania

## Background

Chronic hepatitis is an important problem worldwide, associating high morbidity and mortality, and hepatocellular-carcinoma is one of its most severe complications.

Multiple studies have tried to identify biomarkers that would allow an earlier detection of hepatocellular-carcinoma (HCC), compared to imagistic exams. Such biomarkers are represented by alpha-fetoprotein (AFP), des-γ-carboxy prothrombin (DPC), and the lens culinaris agglutinin-reactive fraction of alpha-fetoprotein (AFP-L3).

## Methods

Since January 2014, the National Institute for Infectious Diseases “Prof. Dr. Matei Balş” has implemented a screening program for hepatocellular carcinoma in patients with hepatitis. The program involves a serum panel performed in the Lotus-MED Medical Center, consisting of AFP, DCP and AFP-L3%, performed at two study visits: screening and 48 weeks follow-up. As the study is still ongoing, we present descriptive data derived from the first study visit.

## Results

We have enrolled 120 patients; their mean age was 56.6±12.2 years, and the male-to-female ratio was 1.07:1. The patients were diagnosed with chronic hepatitis B (8.8%), chronic hepatitis C (72.5%), B+D coinfection (9.9%), idiopathic hepatitis (4.4%) and other causes of liver disease (4.4%). Only 65% of patients had cirrhosis, and 8% of them had a diagnosis of hepatocellular-carcinoma.

The mean values for the serum panel tests were 158.95±1419.82 ng/mL (AFP), 11.68±59.20 ng/mL (DCP), and 14.83±21.85% (AFP-L3%).

In the group of patients with cirrhosis, the positive prediction for HCC based on the serum panel tests was 32.20%. 23.53% of the persons with positive prediction already had a diagnosis of HCC, while 92.5% of the persons with negative prediction did not have a history of HCC. The test’s sensitivity for predicting HCC was 57.14%, and its specificity was 74%. The positive predictive value (PPV) was 23.53% and the negative predictive value (NPV) 92.5%.

## Conclusion

Our preliminary results represent the first serum panel predictions for HCC in the population of Romanian patients with chronic hepatitis. However, given the fact that only 65% of patients displayed cirrhosis at the time of testing, the analysis has also been applied outside of its validated range, and we can only rely on the data available for patients with cirrhosis. The patients will be reinvestigated at 48 weeks, and only then will we be able to calculate an accurate sensitivity, specificity, PPV or NPV, as this serum test is thought to predict a patient’s probability of developing HCC over the next 6 months.

